# Marine-Derived Collagen and Chitosan: Perspectives on Applications Using the Lens of UN SDGs and Blue Bioeconomy Strategies

**DOI:** 10.3390/md23080318

**Published:** 2025-08-01

**Authors:** Mariana Almeida, Helena Vieira

**Affiliations:** CESAM—Centre for Environmental and Marine Studies, Department of Environment and Planning, Campus Universitário de Santiago, University of Aveiro, 3810-193 Aveiro, Portugal

**Keywords:** biobased products, biotechnology, sustainability, circular economy, marine biomass, biopolymers

## Abstract

Marine biomass, particularly from waste streams, by-products, underutilized, invasive, or potential cultivable marine species, offers a sustainable source of high-value biopolymers such as collagen and chitin. These macromolecules have gained significant attention due to their biocompatibility, biodegradability, functional versatility, and broad applicability across health, food, wellness, and environmental fields. This review highlights recent advances in the uses of marine-derived collagen and chitin/chitosan. In alignment with the United Nations Sustainable Development Goals (SDGs), we analyze how these applications contribute to sustainability, particularly in SDGs related to responsible consumption and production, good health and well-being, and life below water. Furthermore, we contextualize the advancement of product development using marine collagen and chitin/chitosan within the European Union’s Blue bioeconomy strategies, highlighting trends in scientific research and technological innovation through bibliometric and patent data. Finally, the review addresses challenges facing the development of robust value chains for these marine biopolymers, including collaboration, regulatory hurdles, supply-chain constraints, policy and financial support, education and training, and the need for integrated marine resource management. The paper concludes with recommendations for fostering innovation and sustainability in the valorization of these marine resources.

## 1. Introduction

Both collagen and chitin of marine origin are biodegradable and biocompatible, with a range of properties that make them suitable in several areas such as biomedical, pharmaceutical, cosmetic, agriculture, and environmental [1], and as alternatives to synthetic polymers [2,3]. Chitin has particular utility in biomedical, environmental, and agricultural fields [3] while collagen, as the primary fibrous protein in the extracellular matrix and connective tissues of animals, plays a crucial role in the development of biomedical products [4]. Several of these uses overlap, while co-application in biomedical hybrid materials has demonstrated synergistic effects, including improved mechanical performance [5] and biological activity [6].

Growing interest for sustainable products and solutions is increasing attention on marine-derived materials like collagen and chitin/chitosan. This trend potential underscores a significant market potential; for example, some reports mention that the chitin, chitosan, and derivatives market was valued at USD 4.01 Billion in 2023 and is projected to reach USD 12.12 Billion by 2032; the marine collagen market is projected to grow from USD 20.49 billion in 2025 to USD 35.43 billion by 2035 [7,8].

In the case of collagen, the global market is particularly driven by the rising awareness of its health benefits, especially among the aging population—for example, its use in supplements aimed at improving skin health and joint function [9]. In agriculture, chitin-based materials are gaining increasing attention for sustainable crop production, serving as natural biopesticides and plant growth enhancers [10].

Blue bioeconomy is an emergent subsector of Blue Economy, encompassing several industries exploring and focusing on biobased products and processes. Blue Economy itself is a wider concept that denotes the use of ocean resources for economic growth, social well-being, and environmental health. In this subsector, biotechnological and biomanufacturing innovations based on marine organisms, such as microorganisms, algae, and invertebrates, can create novel pathways for the commercial exploitation of marine biomass [11,12]. Among these, biopolymers such as collagen and chitin derived from fish and invertebrates exhibit properties that position them as resources relevant in the transition toward a circular and bioeconomy [13,14].

The circular-economy concept, on the other hand, promotes the minimization of material input, waste, emissions, and energy use during the lifecycle of products, while bioeconomy helps reduce the reliance on nonrenewable and unsustainable resources and bear potential for regional economic development [15]. The two concepts coupled together are paramount to several strategic development plans across the globe, and in Europe in particular, such as the Circular Bioeconomy Joint Union partnership, the Biotech and Biomanufacturing Initiative, the upcoming Bioeconomy Strategy 2025, or the Green deal and Fit for 55 strategies, to name a few. Sustainability (and autonomy) is the overarching goal—ensuring that this economic development fosters social and environmental sustained sustainability [16].

To fully harness the potential of marine-derived polymers towards practical applications, a robust innovation pipeline needs to be established. However, while scientific research on both topics is strong, the innovation pipeline remains fragmented and underdeveloped [17].

This review aims to provide an overview of marine-derived collagen and chitin/chitosan, covering their sources, extraction, applications, innovation trends, and policy challenges in the context of the UN SDGs and Blue bioeconomy frameworks, highlighting how these contexts can shape their development and applications. First, it will summarize current research trends. It will then contextualize these developments within the framework of sustainable development, particularly the UN2030 Agenda and its 17 SGDs and relevant European Union policies, mapping both direct and indirect initiatives linked to marine biomass valorization. Finally, the review will examine the main bottlenecks hindering the growth of marine collagen and chitin/chitosan value chains in the EU and will propose areas that require further work to enable their sustainable development.

## 2. An Overview of Marine-Derived Collagen and Chitin/Chitosan: Sources and Properties

Marine-derived polymers such as collagen and chitin/chitosan differ in their biochemical composition and structural characteristics, which in turn influence their functional and application profiles across various fields. Table 1 outlines key sources of marine collagen and chitin/chitosan, highlighting their distinct properties and potential uses.

**Figure 1:**
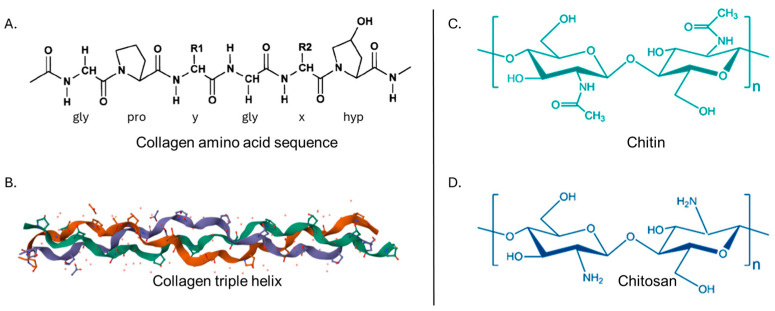
Structure of collagen, chitin, and chitosan. (**A**) Primary amino-acid sequence of Type I collagen, illustrating the repeating Gly-X-Y, where X and Y are often Pro and Hyp, respectively (modified from [22], under CC BY 4.0.); (**B**) three-dimensional model of collagen, illustrating the triple helix (generated with RCSB PDB (rcsb.org)); (**C**,**D**) chemical structures of chitin and chitosan, respectively (modified from [23], under CC BY 4.0.).

Collagen accounts for about 30% of total protein in the human body, and it has a unique triple-helical structure made of three α polypeptide chains, coiled around each other. The α chain’s primary structure consists of repeating Gly-Xaa-Yaa triplets, typically glycine, proline, and hydroxyproline [18] (Figure 1A,B).

Collagen has seen growing interest in its marine-derived form as an alternative to collagen obtained from terrestrial animals [19]. It is commonly derived from fish skins and scales, jellyfish, and sea cucumbers [20,21]. Collagen from fish presents similarities with Type I collagen, the most abundant and bioavailable form in the human body, while collagen from marine invertebrates is more diverse and often exhibits unique structural features, such as variations in amino-acid composition and fibril organization [20,21].

Chitin and its deacetylated derivative, chitosan, are a family of linear polysaccharides composed of varying amounts of (β1→4) linked residues of N-acetyl-2 amino-2-deoxy-D-glucose (glucosamine, GlcN) and 2-amino-2-deoxy-D-glucose (N-acetyl-glucosamine, GlcNAc) residues [24]. It is found in the skeletons of crustaceans and molluscs, and is often processed into chitosan to improve its solubility [24]. The presence of N-acetyl amino groups in chitin facilitates diverse chemical modifications, enabling the development of materials with tailored functionalities [25] (Figure 1C,D).

In recent years, significant scientific advances have propelled the research of marine-derived collagen and chitin/chitosan [17,21,24,26]. Traditionally, isolation methods have relied on conventional chemical-intensive based methods; however, growing environmental concerns have prompted a shift toward more sustainable approaches. Other techniques—such as enzymatic hydrolysis, bacterial fermentation or organic acid-mediated extraction (e.g., citric or lactic acid), supercritical carbon dioxide, or the use of green solvents, such as Deep eutectic solvents (DESs) and ionic liquids (ILs) as alternatives to stronger acids, and energy efficient methods such as microwave assisted extraction, autoclave treatment, ultrasonic-assisted extraction, applied for both collagen and/or chitin, improving yield and reducing solvent/energy use—have demonstrated promising potential in reducing chemical waste and minimizing environmental impact [27,28,29,30,31,32].

Research on marine-derived collagen and chitin/chitosan is primarily driven by biomedical, therapeutic, and pharmaceutical applications, with a high number of studies focused on isolation, characterization, and bioactivities [17]. Beyond the biomedical field, there is growing diversification of the food and nutraceutical sectors for both biopolymers [17,26]. Collagen is also being explored in cosmetic applications, while for chitin/chitosan, there is also a relevant number of publications addressing its use in wastewater treatment, feed, and agricultural applications [17].

Marine-origin collagen has garnered particular interest in the health arena, owing to its biocompatibility with human tissues, lower immunogenicity compared to terrestrial sources, and favorable scaffold-forming properties. The triple helical structure of marine collagen supports superior scaffold formation, facilitating cellular adhesion, proliferation, and differentiation—critical features for tissue engineering and wound healing [33,34]. Marine collagen hydrolysates and bioactive peptides have also demonstrated high bioactivity profile, such as antioxidant, antimicrobial, antihypertensive, and anti-inflammatory properties, making it highly promising for nutraceutical and pharmaceutical applications [35]. For instance, marine collagen-derived peptides can act as effective natural antioxidants, supporting skin health and protecting against oxidative stress [36]. In addition, its high adsorption capacity, moisturizing properties, and ability to improve skin elasticity make collagen a highly valuable ingredient in cosmetics [36,37].

For chitosan, the primary areas of scientific interest are similar to those of marine collagen, focusing on biomedical, pharmaceutical, and food-related applications. In cosmetics, chitosan nanoparticles act as a delivery system for active substances to the skin, enhancing the delivery and stability of active ingredients in cosmetic formulations [38,39].

Additionally, emerging research areas include environmental applications such as water treatment, as well as more diversified fields like agriculture and feed [17].

Key properties of chitin, chitosan, and its derivatives also include biological activities including antitumoral, antimicrobial, antioxidant, and anti-inflammatory activities, which could be used as therapeutic polymers [40]. Chitosan’s antimicrobial and antioxidant activities and film-forming properties further enhance its utility in food preservation [40].

Due to their biocompatibility, biodegradability, low toxicity and non-immunogenicity, they are highly valuable in tissue regeneration, such as wound healing [25,41].

Beyond health-related uses, chitosan’s chelating capacity allows it to bind heavy metals and dyes, making it a potential biodegradable coagulant/flocculant in wastewater treatment [42]. In agriculture, chitosan acts as a natural biostimulant and biopesticide, promoting plant growth and enhancing resistance to pathogens. It is also increasingly used in animal feed for its immunomodulatory and growth-promoting effects [42]. Nanotechnology-derived chitosan and derivatives also promise highly sophisticated biomedical and environmental applications such as biobased electric devices [43] and tumor-targeting drug-delivery systems [41].

The intellectual property landscape reflects the focus on enhancing extraction efficiency and developing sustainable practices and applications in health and food areas, which dominate patent filings, while environmental applications like water treatment are underrepresented in patents. The emphasis on extraction methods and their uses reflects ongoing innovation and technological progress. However, this also reveals a gap between scientific advancements and their commercial application, suggesting untapped potential in areas like environmental sustainability [17].

## 3. UN SDG Lens Applied to Marine-Derived Biopolymers

### 3.1. Marine-Derived Collagen and Chitin Sustainable Uses

**Figure 2:**
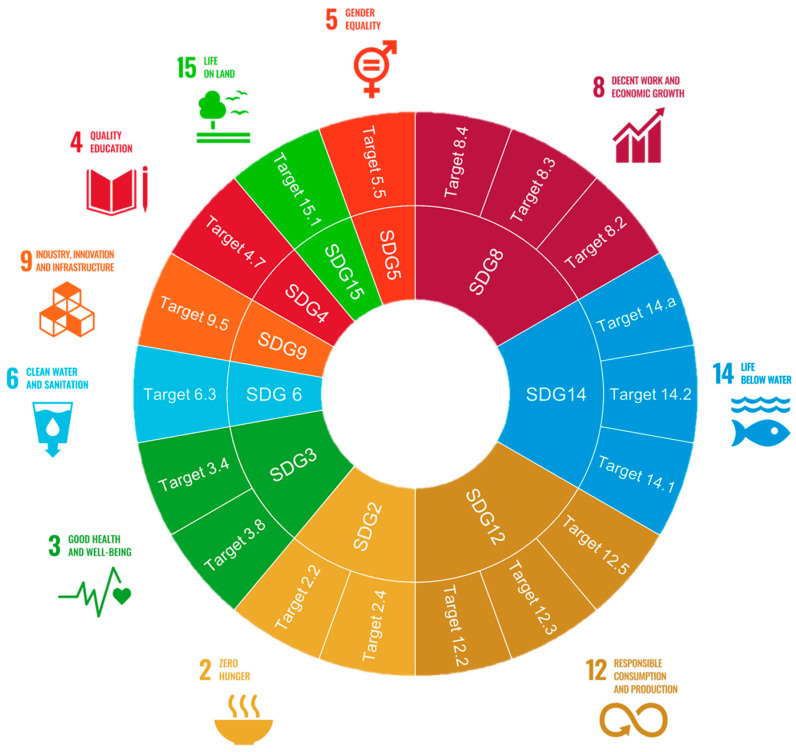
Sustainable Development Goals (SDGs) and targets supported by the development and use of marine-derived collagen and chitin identified through their main application areas—health, food, wellness, and environment [44].

The use of marine-derived collagen and chitin/chitosan aligns closely with the United Nations SDGs [44], as these biopolymers offer sustainable, biodegradable alternatives to synthetic materials while promoting responsible resource use. Overall, the adoption of marine-derived biopolymers plays a significant role in promoting sustainable development across ecological, economic, and social dimensions, contributing, directly or indirectly, to numerous goals and supporting a broad range of sustainability targets [44] (Figure 2, Table 2 and Table 3).

#### 3.1.1. Uses in Health, Agriculture, and Industry

The processing of these polymers is well aligned with SDG 3 (Good Health and Well-Being). Marine-derived collagen and chitosan exhibit bioactive properties such as antimicrobial, anti-viral, antioxidant, wound healing, and tissue-regeneration effects, making them valuable in biomedical applications (e.g., wound dressings, scaffolds, drug-delivery systems) [46,47,48,49], and nutraceutical supplements [22,48,50] and therefore also addresses the ambition of Target 2.2 (addressing global nutrition gaps) [44]. Moreover, the marine collagen tissue-engineered vascular grafts and the hypolipidemic and anti-obesity effects of collagen peptides can contribute to Target 3.4 in the reduction of premature mortality from non-communicable diseases by improving treatment outcomes [51,52,53]. Furthermore, the mucoadhesive properties of chitosan make this polymer a vehicle for mucosal vaccine delivery against infectious diseases [54] and therefore contribute to Target 3.8 by offering alternatives for essential medicines and vaccines.

Beyond biomedical uses, marine chitin and chitosan have garnered attention for their effects in agriculture, including as biopesticides, plant-growth promoters, and soil-health enhancers [17]. Their ability to act on plant pathogens and as stimulants of plant growth [10,55] make them an alternative to synthetic agrochemicals. This expands their relevance to SDG 2 (food security, improved nutrition, and promote sustainable agriculture), particularly Target 2.4 [44], by contributing to sustainable food production systems and enhancing the resilience of agricultural practices. Additionally, their role in reducing chemical-pesticide dependency supports SDG 15 (Life on Land) by minimizing terrestrial ecosystem degradation and negative effects on biodiversity (Target 15.1) [44]. The use of chitosan adsorbent properties in water-purification technologies links to SDG 6 (Clean Water and Sanitation), particularly Target 6.3 [44], by facilitating pollutant removal and water-quality improvement [56].

Ongoing advances in extraction technologies, processing, and technological development continue to expand the potential of marine collagen [22,28,57] and chitin/chitosan [58,59] and therefore align with SDG-9 (Industry, Innovation, and Infrastructure), Target 9.5, by enhancing scientific research and technological innovation.

#### 3.1.2. Sustainability and Circular Economy

The concepts of sustainability and the circular economy aim to conserve natural resources, reduce waste, promote cleaner and more efficient production methods, and support long-term environmental health by maximizing the value derived from materials throughout their lifecycle [16]. Within this framework, the valorization of marine biomass, such as collagen and chitosan derived from fish processing by-products and crustacean shells, offers a sustainable alternative to the direct extraction of primary marine resources and therefore directly supports SDG 14 (Life below Water), by prioritizing the valorization of this biomass over direct extraction from primary marine resources [26], reducing environmental pressures such as overexploitation and pollution. It also aligns with actions to increase scientific knowledge, develop research capacity, and transfer marine technology to improve ocean health [17], aligning in particular with Target 14.a. The development of biodegradable chitosan-based materials can also mitigate plastic pollution, contributing to Target 14.1 (prevent and significantly reduce marine pollution of all kinds) [60].

Furthermore, the use of collagen and chitosan from marine by-products promotes sustainable resource utilization and waste minimization, directly supporting SDG 12 (Responsible Consumption and Production) and in particular Target 12.2 (achieve the efficient use of natural resources), 12.3 (halving per capita global food waste and reducing food losses along production and supply chains) and Target 12.5 (substantially reducing waste generation through prevention, reduction, recycling, and reuse) [61]. It is also aligned with Target 14.2 (sustainably manage and protect marine and coastal ecosystems), by supporting circular- and bioeconomy approaches that promote the sustainable management of ocean resources [62].

These initiatives also align with SDG 4 (quality education), through the promotion of lifelong learning opportunities through capacity building, skills development (Target 4.4) [44], specifically tailored to the blue economy sector [63] and in the promotion of education for sustainable development (Target 4.7), equipping individuals with the knowledge and skills needed to support sustainability goals.

In a broader context, the valorization of these marine biopolymers contributes to SDG 8 (Decent Work and Economic Growth), by creating new value chains and new jobs, and stimulating entrepreneurship, supporting Targets 8.2 (improve economic productivity through diversification and innovation), 8.3 (promote policies for jobs, entrepreneurship and innovation), and 8.4 (improve resource efficiency in consumption and production). In particular, the ten Ocean Decade Challenges are part of the United Nations Decade of Ocean Science for Sustainable Development (2021–2030) and articulate the most immediate priorities for the Ocean Decade. Challenge 4 calls for the creation of a sustainable and equitable ocean economy, emphasizing the importance of fostering economic inclusion, particularly in coastal communities [64]. In this context, the use of these polymers supports socio-economic development by creating value chains and developing industries around these products: coastal communities can create new jobs, stimulate entrepreneurship, integrate scientific innovation with local resource management, and diversify the local economy beyond traditional sectors like fisheries or tourism [17].

In addition, integrating gender equality into this development aligns with Target 5.5 [44] by promoting women’s full and effective participation, as well as equal opportunities for leadership [65].

### 3.2. The EU Blue Bioeconomy and Challenges and Opportunities for Marine-Derived Biopolymers

Recent advances in the Blue bioeconomy underscore the strategic importance of marine-derived biotechnological innovations for achieving sustainability and circularity goals [11,12]. EU policy initiatives—such as the upcoming revision of the EU Bioeconomy 2025 Strategy, the EU Ocean Pact [66] the European Green Deal, the Circular Bio-based Europe Joint Undertaking, and the Circular Economy Action Plan—provide a policy and funding landscape that emphasizes the role of the ocean and its resources to the Union and a vision of a sustainable path needed to foster a new economic paradigm anchored in sustainable models, including waste reduction, resource efficiency, circularity, and the sustainable use of biological resources. At more local and regional level, national initiatives, such as the Nature Restoration Law national directives implementation, or even national dedicated ocean or bioeconomy strategies, may influence or shape the development of the markets and technologies related to these marine polymers.

On the other hand, recent market forecast projections for these marine-derived polymers are estimating a doubling or tripling market-value opportunity [11,12] and, despite the enormous development opportunities these projections may bring, they also entail concrete sustainability and value-chain management challenges that it is important to tackle.

**Figure 3:**
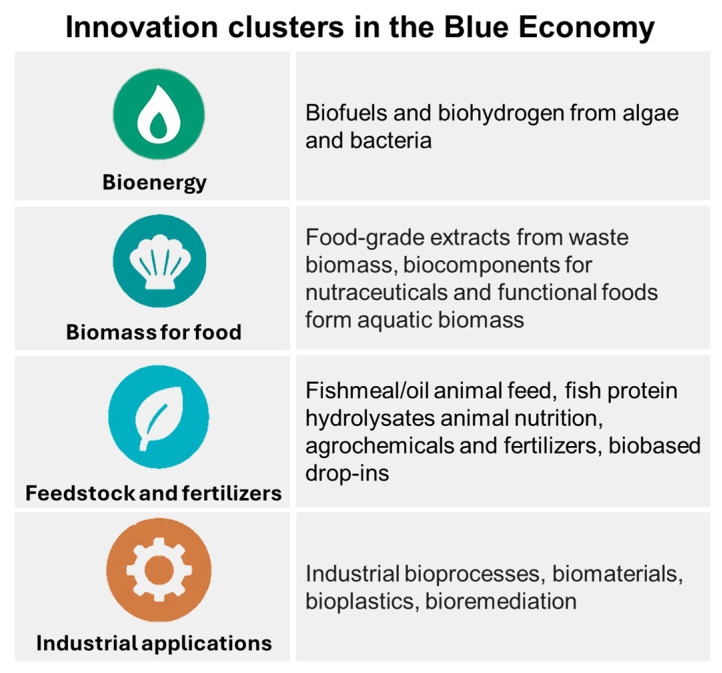
Clusters of Blue biotechnological innovations identified by Pakseresht et al. [12]: (1) bioenergy from marine biomass, (2) biomass for food, (3) feedstock and fertilizers, and (4) industrial applications. These clusters reflect the valorization potential of marine-derived resources and their alignment with sustainable and circular business model patterns.

Pakseresht et al. [12] identified four core innovation clusters in the Blue biotechnology innovation arena (bioenergy, feedstock, and fertilizers, biomass for food, and industrial applications), each representing different resource applications, technological developments, and potential business models that can extend beyond economic value to address environmental and social values (Figure 3). This categorization was achieved through a comprehensive scoping review of the global scientific literature, analysing emerging trends and technologies, especially in the context of business and management.

The cluster on the production of feed and fertilizers focuses on transforming marine waste, such as fishery and shellfish discards and processed by-products, into valuable inputs like animal feed, fish protein hydrolysates for animal nutrition, or agrochemicals fertilizers. Activities in the biomass for food cluster utilize marine organisms such as marine waste, algae, and invertebrates from mariculture to produce food products, nutraceuticals, and functional ingredients. The industrial applications cluster encompasses the development of bioplastics, biomaterials, and environmentally friendly materials derived from marine biomass such as algae, microorganisms, and marine waste into a wide variety of products with applications in a vast number of industries, from furniture and bioconstruction, to beverages, water treatment, or even household products.

In the last decade, marine invertebrates have been increasingly investigated as potential sources of novel biomaterials, although issues related to sustainability and circularity need further research [21]. In an era where biomass sourcing and sustainability is a paramount concern for each nation, in particular in the EU, fostering marine by-products or marine-related industries’ waste streams’ innovative usages, alongside the sustainable production of dedicated aquatic biomass, and using the cascading principle to support the zero-waste concept, can be the key for the quest for autonomy that many nations are aiming for [67] (CBE-JU SC recommendations, 2024).

However, some applications are already well developed and cost-effective, while others offer high potential or a significant market impact but still face challenges related to regulation, regional demand, biomass-sourcing limitations, or the need for substantial research and development [12]. According to the same authors, the feedstock and fertilizers (like fishmeal and organic fertilizers) cluster-related innovations are mature with moderate market value and manageable costs. On the other hand, biomass for food (novel marine foods, functional ingredients) shows strong growth and impact potential but depends on regional markets and tight regulation, while industrial applications (bioplastics, cosmetics, pharmaceuticals) offer the highest market impact but still need heavy R&D and investments.

**Figure 4:**
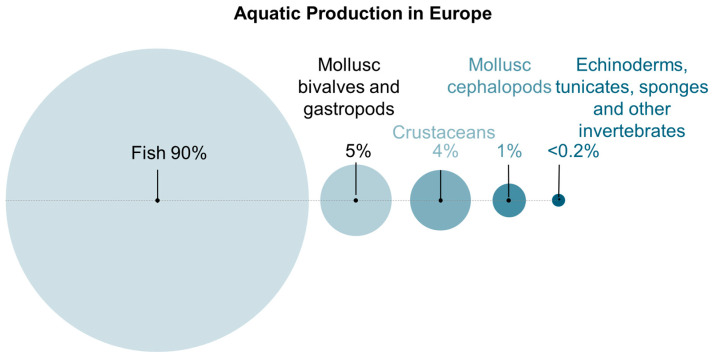
Proportion of total aquaculture and capture production of aquatic species by animal group in Europe. Data obtained from the Food and Agriculture Organization of the United Nations [69].

**Figure 5:**
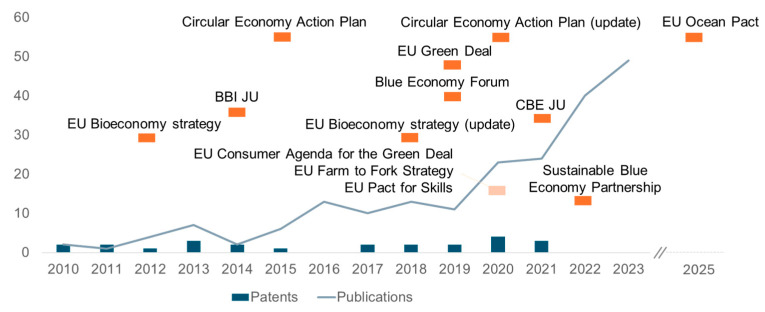
Number of scientific publications and patents related to marine-derived collagen and chitin/chitosan in Europe (1990–2023) Data adapted from [17]. European policy strategies directly or indirectly linked to the Blue bioeconomy.

These challenges were also highlighted within the EU context, where, despite its unique and abundant marine biomass resources, and active research in this field (Figure 4 and Figure 5), there remains a need for tailored policies and investments to turn European Blue biotechnology potential into tangible results [17]. Although EU policies may have improved the development of ocean-related economies, with research in marine-derived collagen and chitin/chitosan increasing in Europe in the last decade, innovation and market translation still remains weak (Figure 3), as evidenced by lower levels of intellectual property filings compared to other regions such as China and USA [17,68]. The translation of this research into innovations is currently dominated by Asia, with countries such as China, Japan, and South Korea leading in both publication output and patent activity related to marine-derived polymers [17]. In contrast, European countries show a relevant academic presence and outputs, with some countries dominating even in the world arena (like Norway or Portugal), but show a relatively weaker representation in intellectual property filings [17].

This suggests that there are limitations or inadequate frameworks in these regions for technology transfer and effective valorization of these resources, with underlying reasons for these limitations—such as technical and strategic challenges—already identified across multiple areas of the Blue bioeconomy [15,17,63].

The main technical challenges of the use of these marine biopolymers are, among others, biomass availability and seasonality, low extraction yields and composition variability due to seasonality, high chemical usage, and limited scalability [70]. There are also reports on challenges in technological access and innovation. One possible approach to reducing some of these challenges is AI-driven optimization (e.g., in extraction efficiency, data analysis) to improve efficiency, reduce waste, and enable scalable production systems [71].

In addition to technological issues, regulatory constraints pose significant hurdles, especially in some regions and in some markets. Regulatory complexity, such as governing safety, quality, labelling, product consistency, source certification, and differences in the requirements depending on the source of the biopolymer and the intended application of the final product are major bottlenecks [29,72]. Specifically, EU regulations concerning novel food, cosmetics, biomedical, pharmaceutics claims and usages, and management of the discard and by-catch from fisheries and aquaculture, underscore the necessity of complying with these regulations at various stages—from raw-material collection to final-product manufacturing [13,73], and this can function as an added burden when competing in a global and unequally demanding world. Environmental impact assessments, and bureaucratic delays were also identified as serious innovation blockers at a regional level [74].

Barriers also persist in technology transfer, as insufficient emphasis on intellectual property (IP) management in the research processes [17,63,72] coupled with low levels of IP literacy leads to lower patenting activity in Europe. Challenges in IP management, costs, and the difficult and specialized patenting process can delay the time it takes for new products to reach the market. Additionally, patent protections are limited to certain territories and durations, and the initial embargo on the publication of recent patent applications delays knowledge dissemination and increases challenges in effectively managing IP within the Blue bioeconomy sector [74].

A lack of education and training has also been identified as a barrier. Policies exist to promote the updating of educational curricula and training programs to integrate bioeconomy concepts and provide practical, industry-specific training, but they are criticized for lacking specificity and not adequately addressing the unique training needs of smaller enterprises and primary producers [75]. Recent policy initiatives to address capacity building, such as the EU Pact for skills, may reduce this gap in the alignment of innovation and IP generation goals.

Raising awareness of the potential of the Blue bioeconomy and marine biopolymers among industry stakeholders, fishermen, the public, and consumers has also been identified as a solution to overcoming these barriers [74]. In particular, strengthening collaboration with manufacturers and product developers is crucial to ensure that research outcomes align with market demands. Similar to the growing recognition of the advantages and sustainability related to biodegradable polymers, increased awareness of these links to the ocean could boost demand for marine biopolymers-derived products, such as smart food packaging and health supplements, and could act as a driver for the technological development of the Blue bioeconomy [68].

Fostering collaboration is continuously pointed out as essential to engage the industry in research and development. Academia–industry collaborations through innovation clusters and co-funded projects can establish critical mass, facilitate knowledge exchange, accelerate knowledge transfer, facilitate political and financial support, and produce the most creative and innovative results to address important societal challenges [74]. However, the current approaches to collaboration are considered too broad, lacking the specific operational details necessary to effectively engage diverse stakeholders and tailor initiatives to the unique regional and/or sectorial contexts and needs of different industries within the bioeconomy [75]. Community-driven approaches may help reduce the few and/or fragmented bio-businesses, low collaboration, and lack of awareness by promoting early, inclusive engagement and regional cooperation. Integrating these strategies into development plans enhances the effectiveness and sustainability of Blue bioeconomy initiatives [15,74]. These collaborations are essential, particularly in areas where there is resistance or hesitation to participate. Building strong partnerships helps to build trust, share knowledge, and demonstrate the benefits of involvement, making these stakeholders more willing to engage in such activities [74].

## 4. Final Remarks

Crustaceans contain approximately 40% meat, with the remaining 60% being inedible [68] and mostly discarded as waste. However, crustacean waste contains chitin, a biocompatible and biodegradable polymer that can be processed into chitosan and used in advanced medical technologies, such as nerve-regeneration conduits (e.g., Reaxon, manufactured by Medovent in Germany [76]), as well as in environmental solutions like water clarifiers (e.g., SeaKlear, produced by Primex in Iceland [77].

Similarly, the fish-processing industry produces a huge amount (ranging from over 25% to 70%) of by-products [78], which can also be valued as a source of collagen to produce high-value products such skin-regeneration and wound-healing biomaterials (for example, Omega3 Wound, manufactured by Kerecis in Iceland [79]).

This growing recognition of the value of marine bioresources and their biomass and processes lies in the comprehensive exploration of their diverse applications across multiple areas, generating new market opportunities and fostering new sustainable models of economic development like the bioeconomy. However, potential weaknesses in Europe for technological innovation may include the need for further in-depth analysis of the specific challenges within the marine-derived collagen and chitin/chitosan value chains. As we and several authors point out, the bottlenecks for Blue bioeconomy development in Europe, such as persisting knowledge gaps, environmental sustainability concerns, regulatory complexity and uncertainty, technological scaling needs and gaps, high monetary and time investment needed, disconnected cross-collaboration frameworks, and persisting challenges for market acceptance of biobased products, are equally applicable to marine collagen and chitin supply chains [17,74,80]. In particular, a significant hurdle remains in the uptake of marine biopolymer research by industry, particularly among manufacturers and product developers. Many companies face uncertainty regarding the scalability, cost-efficiency, lack of standardized quality, and regulatory pathways across several application areas and reliable supply chains, particularly in raw-material limitation, when using marine-derived ingredients [22,81]. In this regard, the contribution of aquaculture-sourced raw materials and selective processing may offer promising alternatives to reduce dependency on natural seasonal biomass while ongoing ecosystem monitoring can help ensure that any harvested sources are managed sustainably [13,26]. These challenges highlight key priorities that emerge for advancing the development of marine collagen and chitin/chitosan value chains. First, advancing sustainable and scalable extraction methods—particularly those that reduce environmental impact through green or enzymatic processes—remains essential [82]. Equally important is the establishment of standardized protocols for quality consistency, which would facilitate both regulatory approval and industrial uptake [22]. In parallel, there is a clear need for comprehensive life-cycle and economic and social analysis to substantiate sustainability claims and guide investment decisions, which are currently very limited and often purely academic [13], for example, comparative life-cycle analysis or environmental-impact assessment of marine-derived versus terrestrial-derived materials. Finally, addressing regulatory complexities, having an understanding of market opportunities, and analysing and improving market acceptance will require supportive policy frameworks that reduce perceived risks, promote investment in sustainable innovations, and improve the transdisciplinary collaboration among researchers, industry stakeholders, and policymakers. Together, these directions can help close persistent gaps and advance the integration of marine-derived polymers into a sustainable European Blue bioeconomy.

Although five years remain to achieve the UN Agenda 2030 Sustainable Development Goals, recent monitoring of all SDGs in the EU context highlights ongoing challenges. The 2024 Eurostat report emphasizes that while waste generation has slightly decreased, the circular use of materials is not increasing quickly enough to meet long-term targets [83]. This signals a need for accelerated efforts to scale up sustainable-resource use and waste reduction across all sectors—including the marine bioeconomy [83]. Other SDGs are also at risk of not being reached and the low investment in SDG14 is also of particular relevance to this work and context. [84] For example, Northern European regions—such as Norway and the Baltic Sea—have relatively advanced marine biotechnology sectors, with approaches combining research, industrial application, and regulatory frameworks that encourage the sustainable valorization of marine biomass and support circular-economy goals. In comparison with the Mediterranean, countries in this region often face challenges related to fragmented regulatory structures, limited infrastructure, or underexploited marine-biomass streams [85].

Beyond Europe, in countries like Brazil, marine biotechnology is advancing, supported by national research initiatives, but challenges remain in translating academic research into clinical products, often due to gaps in regulatory frameworks, and the need of training programs to foster coordination between academia, industry, and government [84].

Through continued scientific research, innovation-driven policies, and strategic cross-sectoral collaboration across research, industry, administration, legal services, market analysis, and public or social sectors, these polymers can play a role in the EU Blue bioeconomy and in supporting the neutrality ambitions of the European continent. In fact, the EU Blue Economy Report 2025 highlights that Blue Biotechnology—driven by marine-derived innovations like these polymers—grew by 19% in gross value added and 18% in turnover in 2022, reflecting its increasing role in advancing high-value, sustainable sectors across multiple industries, such as pharmaceuticals, biomaterials, and ecosystems [86] Other recent reports from OECD and the UN on ocean-sustainable economy and marine biotech potentially co-substantiate these projections too [87,88].

Their Integration Into high-value supply chains will not only support circularity and green innovation but will also foster economic resilience in coastal communities.

Knowledge transfer and capacity building can also play a crucial role in advancing the bioeconomy of these polymers in other regions of the world, enabling the adoption of sustainable marine biotechnology.

## 5. Materials and Methods

This review analyzes the applications of marine-derived collagen and chitin/chitosan from two perspectives based on their alignment with the SDGs, and their contribution to Blue bioeconomy strategies.

The potential contribution of collagen and chitosan value chains to specific SDGs was identified by examining how IP claimed main application areas—health, food, wellness, and environmental—aligns with the goals and targets outlined in official SDGs documentation [44]. Global progress toward these selected targets, as reported in the UN SDG Report 2024 [45], was indicated using the following classifications: 🟥 Off track, 🟨 Limited progress, 🟩 On track.

The degree of contribution of each marine-derived biopolymer to each SDG was assessed using data from a previous study [17], which analyzed global trends in patents and scientific publications related to these polymers. Specifically, the percentage of distribution of International Patent Classification (IPC) codes—each corresponding to a specific technological field—determined in that study was used as a proxy for each thematic focus contribution. For example, the IPC subclasses A61K, A61P, and A61L are associated with medical, pharmaceutical, and wound-healing technologies, while A23L and A23K relate to food and feed technologies. The sum of these IPC thematic codes provided a distribution per biopolymer across thematic areas. These percentage ranges of IPC occurrence (1.3–25.4% for collagen; 1.1–16.3% for chitin/chitosan) were then divided into three equal-width categories using the equal interval method. This allowed the classification of each domain’s contribution as Strong (🟢), Moderate (🟡) or None/residual (🔴) levels across thematic fields. For the more general SDGs impact analysis, an empirical approach was used based on the existing literature and state-of-the-art knowledge, but subject to the researcher’s expertise and theoretical background, to determine the degree of contribution to each target. The same color code with round circles was used.

A subset of data was also extracted from the study [17] to report the intellectual property and academic research trends in Europe. In the original study, the patent search was conducted using the WIPO PATENTSCOPE database, employing keyword queries applied to titles and abstracts, and refined through inclusion/exclusion criteria based on marine origin and relevance. The scientific publications were retrieved from the Scopus database using the same keyword logic and were screened by title, abstract, and full text when necessary to verify marine origin and application focus. The study identified a total of 3515 patent documents (2372 for collagen and 1143 for chitin/chitosan) and 877 scientific publications (419 for collagen and 458 for chitin/chitosan) from 1990 to 2023. For the purpose of this review, only data concerning European countries were selected, resulting in a final subset of 24 patents and 205 publications.

## Figures and Tables

**Table 1:** Comparative overview of collagen and chitin/chitosan: structure, sources, properties, applications, and commercial trends.

	Marine Collagen	Marine Chitosan (from Chitin)
Chemical Structure 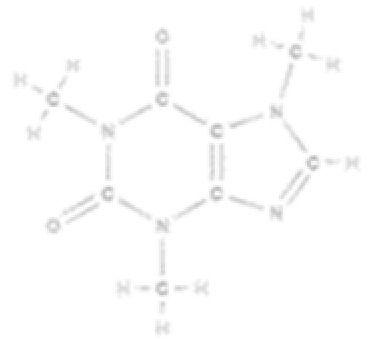	- Triple-helix of 3 α polypeptide chains (Gly-Xaa-Yaa)—rich in glycine, proline, hydroxyproline	- Linear polysaccharide (β1→4)-linked GlcN and GlcNAc units—derived from chitin via deacetylation
Sustainable Sources 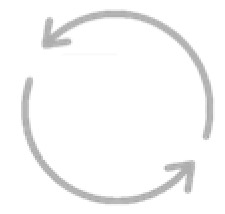	- Fish skin and scales - Jellyfish - Sea cucumbers	- Crustacean and mollusc skeletons
Sustainable Processing/Extraction 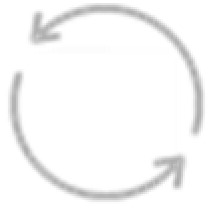	- Enzymatic hydrolysis- Green solvents - Fermentation-Ultrasound-assisted extraction	- Similar sustainable extraction methods being explored
Properties 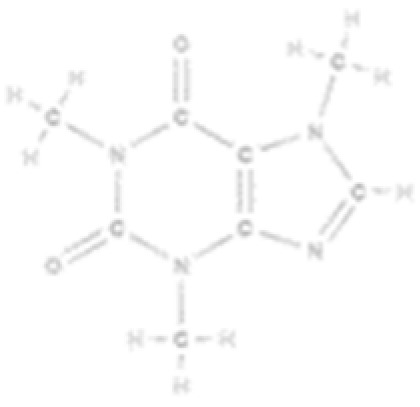	- Biocompatible; bioactive profile; low immunogenicity; scaffold-forming	- Biocompatible; biodegradable; non-toxic- antimicrobial; film-forming
Key Biomedical/Pharmaceutical Applications 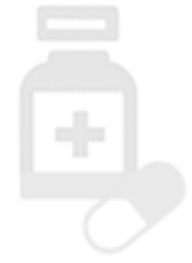	- Tissue engineering - Wound healing - Antioxidant, antimicrobial, anti-hypertensive uses	- Tissue regeneration - Wound healing - Antitumoral, antimicrobial, antioxidant uses - Nanotechnology (biosensors, drug delivery, bioelectronics)
Cosmetic Applications 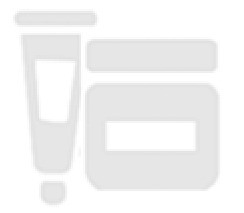	- Skin health - Moisturizer - Anti-aging - Elasticity improvement	- Film-forming for protective coatings (indirect cosmetic potential) - Advanced delivery systems to the skin
Food/Nutraceutical Uses 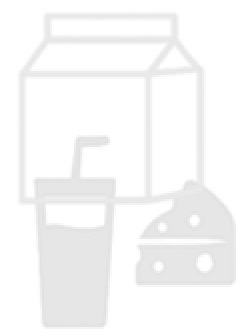	- Nutritional supplements - Functional foods	- Food preservation (antioxidant and antimicrobial films) - Food additives
Environmental Uses 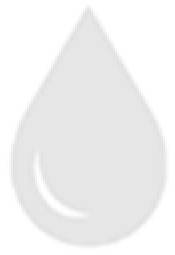	Limited (potential in sustainability but underexplored commercially)	- Wastewater treatment (chelation, flocculation) - Agriculture (biopesticide, biostimulant) - Animal feed (growth promoter)
Commercial Trend & Patents 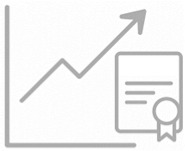	- Focused on health and food sectors	- Similar focus to collagen - Environmental uses underrepresented in IP landscape

**Table 2:** Contribution of collagen and chitin/chitosan value chain and related IP to selected Sustainable Development Goals (SDGs) and targets organized by market application. Square color icons (🟥 Off track, 🟨 Limited progress, 🟩 On track, ⬜no data) indicate the global status of each target based on the UN SDG Report 2024 SDG Progress [45]. Rounded color icons (🟢 Strong, 🟡 Moderate, 🔴 None or residual, **⚪** not estimated) indicate the degree of contribution collagen and chitin/chitosan to each target.

Area	Applicable SDGs	Applicable Targets	Progress	Collagen	Chitin
				Potential Contribution	
Section 1—Application-Driven SDG Contributions					
Healthcare 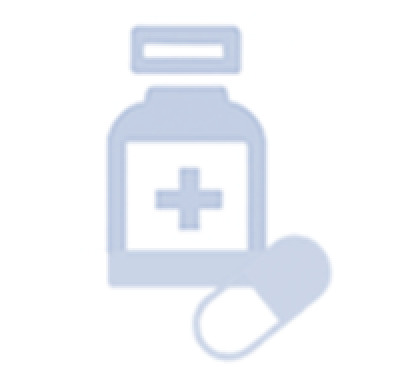	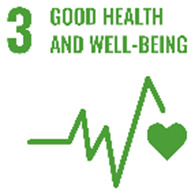	3.4 reduce premature mortality from non-communicable diseases	🟨	🟢	🟢
		3.8 access to safe, quality, affordable medicines and vaccines	🟥	🟢	🟢
Cosmetics 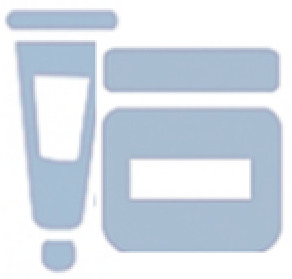	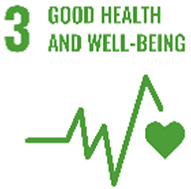	3.9 reduce health impact from hazardous chemicals and air, water, and soil pollution and contamination	🟩	🔴	🔴
Food & Nutrition 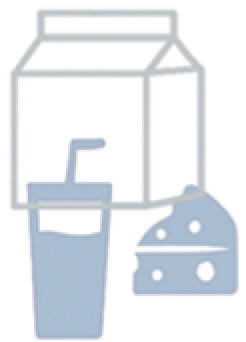	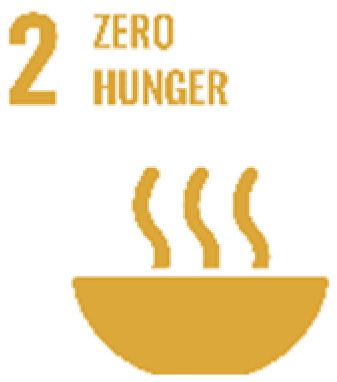	2.2 address nutritional needs	🟥	🟢	🟡
Water treatment 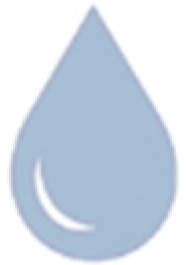	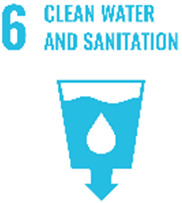	6.3 improve water quality by reducing pollution	🟨	🔴	🟡
Agriculture 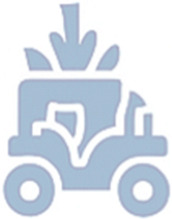	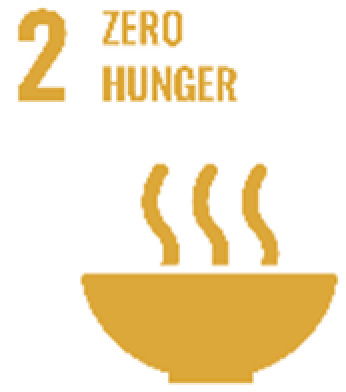	2.4 ensure sustainable food-production systems	⬜	🔴	🟢
	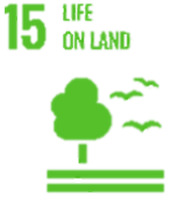	15.1 ensure the conservation, restoration, and sustainable use of terrestrial and inland freshwater ecosystems	🟥	🔴	🟢

**Table 3:** Other SDGs including 14, Empirical and Potential Contribution of collagen and chitin/chitosan value chains to selected Sustainable Development Goals (SDGs) and targets. Square color icons (🟥 Off track, 🟨 Limited progress, 🟩 On track, ⬜no data) indicate the global status of each target based on the UN SDG Report 2024 SDG Progress [45]. Rounded color icons (🟢 Strong, 🟡 Moderate, 🔴 None or residual, **⚪** not estimated) indicate the degree of contribution collagen and chitin/chitosan to each target.

Applicable SDGs	Applicable Targets	Progress	Collagen	Chitin
			Potential Contribution	
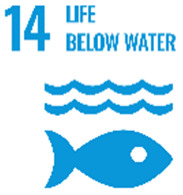	14.1 prevent and reduce marine pollution	🟨	🟡	🟢
	14.2 sustainably manage and protect marine and coastal ecosystems	🟨	🟢	🟢
	14.a increase scientific knowledge, develop research capacity, and transfer marine technology for ocean health	🟥	🟢	🟢
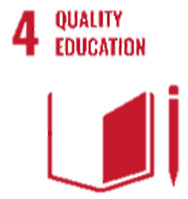	4.4 promotion of lifelong learning opportunities through capacity building, skills development	⬜	🟡	🟡
	4.7 acquire knowledge and skills to promote sustainable development	⬜	🟡	🟡
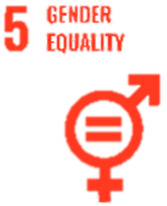	5.5 ensure women’s effective participation and equal opportunities for leadership	🟥	🟡	🟡
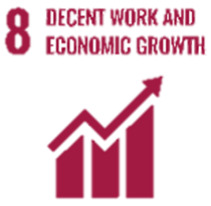	8.2 improve economic productivity through diversification and innovation	🟥	🟡	🟡
	8.3 promote policies for jobs, entrepreneurship, and innovation	🟥	🟡	🟡
	8.4 improve resource efficiency in consumption and production	🟨	🟡	🟡
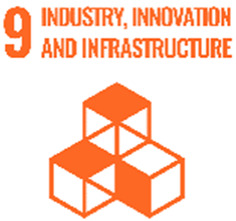	9.5 enhance scientific research, upgrade the technological capabilities of industrial sectors	🟩	🟡	🟡
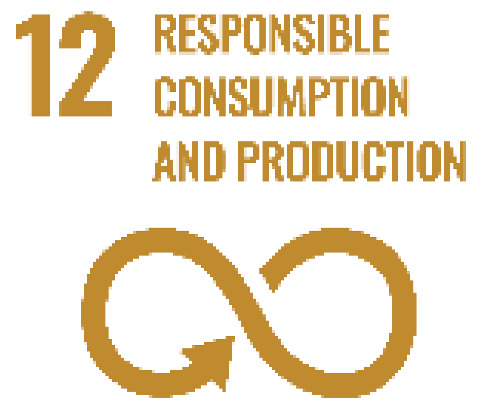	12.2 sustainable management and efficient use of natural resources	🟥	🟢	🟢
	12.3 food losses reduction along production and supply chains	🟥	🟡	🟡
	12.5 reduce waste generation through prevention, reduction, recycling, and reuse	🟥	🟢	🟢
